# Perceived Anxiety is Negligible in Medical Students Receiving Video Feedback During Simulated Core Practical Skills Teaching: A Randomised Trial Comparing Two Feedback Modalities

**DOI:** 10.7759/cureus.7486

**Published:** 2020-03-31

**Authors:** Joanna Matthan, Matthew Gray, Craig I Nesbitt, Lucy Bookless, Gerard Stansby, Alexander Phillips

**Affiliations:** 1 Dental Sciences, Newcastle University, Faculty of Medical Sciences, Newcastle upon Tyne, GBR; 2 Department of Trauma and Orthopaedics, Royal Victoria Infirmary, Newcastle upon Tyne, GBR; 3 Vascular Surgery, Freeman Hospital, Newcastle upon Tyne, GBR; 4 Surgery, Northumbria Healthcare Trust, Newcastle upon Tyne, GBR; 5 Northern Oesophagogastric Unit, Royal Victoria Infirmary, Newcastle upon Tyne, GBR

**Keywords:** anxiety, stress, video feedback, clinical skills, face-to-face feedback, one-to-one feedback, self-assessment, reflection, reflective practice

## Abstract

Introduction

The ability to undertake simple practical procedures is essential for graduating medical students and is typically assessed using simulated models. Feedback is a key component of the learning process in developing proficiency in these key skills. Video feedback (VF) has previously shown promise, however, negative effects of VF-related anxiety on performance have been previously reported. Our aim was to investigate for a difference in participant anxiety between supervised individualised video feedback (SIVF) and unsupervised generic video feedback (UGVF) when undertaking simulated basic practical procedures.

Methods

Undergraduate medical students participating in a clinical skills study to compare UGVF and SIVF completed a Likert scale questionnaire detailing perceived anxiety. During the study, students were recorded performing three basic surgical skills (simple interrupted suturing, intravenous cannulation, urinary catheterisation). Feedback was then provided by one of two methods: (1) SIVF - participant video footage reviewed together with a tutor providing targeted feedback, and (2) UGVF - participant video footage reviewed alone with concurrent access to a generic pre-recorded ‘expert tips' video clip for comparison. Each participant received SIVF and UGVF at least once.

Results

The majority of participants did not find either SIVF (81.7%) or UGVF (78.8%) stressful. Students had a strong preference for SIVF (77.5%) and disagreed that similar ‘face-to-face’ feedback had impaired learning in the past (80.3%).

Conclusion

Medical student-perceived anxiety is negligible when video feedback is employed during simulated core practical skill training.

## Introduction

The ability to undertake basic practical procedures safely and effectively is an essential prerequisite for the graduating medical student. In the United Kingdom (UK), the General Medical Council (GMC) sets the standards for undergraduate learning in Tomorrow’s Doctors (2009) stating that graduates are expected to 'be able to perform a range of therapeutic procedures' including intravenous cannulation, urethral catheterisation and skin suturing, amongst some 24 other practical procedures outlined in the guidance [1]. Additionally, the GMC places significant emphasis on the importance of student-directed feedback, where students are accountable for learning and subsequent performance [1].

Indeed, feedback is vital for medical practitioners [2]. Feedback affords learners the opportunity to identify the gap between actual and desired performance levels and subsequently how that gap can be narrowed to improve performance. Medical students, in particular, have been shown to have increased satisfaction when feedback on their performance is optimised in terms of both quality and quantity [3]. In spite of this, in a national survey of UK-based students across all subject disciplines over nine years, medical students were amongst the least satisfied with feedback provision in terms of punctuality, quality and utility [4]. The continual expectation for educational institutions to strive to improve the quality of feedback, in turn, drives the pursuit of newer feedback modalities, such as the use of video technology in the context of simulated technical skills.

Whilst there are several barriers to learning associated with feedback, emotional distress - or anxiety - may become one that prevents learners from using and accepting the feedback provided to them, especially if the feedback either threatens their self-esteem or is not in line with their own assessment of their ability. Feedback associated with negative emotions may have a long-lasting impact on students’ learning and it has been previously acknowledged that excessive levels of stress may have an adverse effect on performance in a simulation and surgical skills context [5, 6-11].

With these considerations in mind, the relationship of video feedback and participant anxiety was to be examined, with a view to enhancing the provision of feedback during future simulation-based medical education. Specifically, the aim of this study was to investigate for a difference in perceived participant anxiety levels between supervised individualised video feedback (SIVF) and unsupervised generic video feedback (UGVF) when medical students undertook simulated practical procedures.

## Materials and methods

A randomised trial comparing two types of VF was conducted at Newcastle Medical School, UK. Participants were recruited as part of a larger trial to evaluate the impact on two types of feedback [12]. Participants were asked to watch an initial informative video on three practical skills (intravenous cannulation, urethral catheterisation and suturing) and were then invited to perform each of these tasks independently and privately whilst being video-recorded. These skills were identified as skills that were important to learn at the undergraduate level and easily recorded with precise steps required in order to be carried out correctly. Each task was to be completed within a set time-frame, simulating exam conditions.

Participants were given feedback on their video performance via one of two methods to which participants were randomised: they either (i) watched a video-recording of their own performance accompanied by an ‘expert tutor’ (SIVF), who provided suggestions for improvement or (ii) reviewed a video-recording of their own performance unaccompanied, after which they could access a video clip of an ‘expert’ performance of the same skill with generic hints and tips annotations (UGVF).

Five SIVF ‘expert tutors’ (all qualified medical doctors), were available for provision of feedback. The experts were competent in intravenous cannulation, urethral catheterisation and interrupted suturing procedures and were previously unknown to the students. Prior to the onset of the trial, experts received a briefing regarding the logistics of the study and the principles expected for the provision of constructive feedback.

Students were permitted a maximum of seven minutes to perform each task which was identified as the appropriate time that students might be given for a similar task in an objective structured clinical examination (OSCE) scenario, following the standard Newcastle Medical School clinical skills examination format. Subsequent feedback sessions lasted 20 minutes, to ensure there was adequate time for students to review their performance and analyse it either individually or with a trainer, and immediately followed the completion of the task. Each participant experienced SIVF and UGVF at least once, undertaking three tasks in total during the trial day, one for each of the three procedural skills (see Table 1).

**Table 1:** Questionnaire statements to which students were asked to provide a LIKERT response

ITEM NUMBER	STATEMENT	RELATED THEME
1	Receiving feedback from an expert was stressful	Supervised video feedback (DEF)
2	I felt more at ease not having an expert reviewing my performance	DEF vs UVF
3	Reviewing my own performance with an expert video was stressful	Unsupervised video feedback (UVF)
4	I prefer to have face-to-face feedback rather than using other types (e.g. written, unsupervised video)	Overall student feedback preferences
5	Face-to-face feedback has impaired my learning previously due to anxiety	Impact of anxiety on learning

After receiving feedback for their third task, students completed a questionnaire measuring their perceived levels of stress and anxiety with regards to their experience of both SIVF and UGVF. A five-point Likert scale ranging from strongly disagree (1) to neither agree nor disagree (3) and strongly agree (5) was used for five rating statements (see Table 2).

**Table 2:** Median responses to each statement

	ITEM ONE Receiving feedback from an expert was stressful	ITEM TWO I felt more at ease not having an expert reviewing my performance	ITEM THREE Reviewing my own performance with a pre-recorded expert video was stressful	ITEM FOUR I prefer to have face-to-face feedback rather than using other types	ITEM FIVE Face-to-face feedback has impaired my learning previously due to anxiety
Median	1 (strongly disagree)	3 (neither agree nor disagree)	1 (strongly disagree)	5 (strongly agree)	1 (strongly disagree)

 

Written consent was gained from all participants and ethical approval obtained from the Newcastle University Ethics committee. Response frequencies were determined and a Chi-squared test for trend analysis was undertaken to compare responses based on gender and clinical experience (pre-clinical vs. clinical students).

## Results

Of the 71 medical students who participated in this questionnaire-based study, 32 were female, 37 were male and two were participants did not state their gender. Year groups spanning the breadth of the medical degree course at Newcastle were represented by 32 pre-clinical students (years 1 and 2) and 39 clinical students (years 3, 4, 5). Median participant age was 22 (range = 18-37).

Receiving feedback

**Figure 1:**
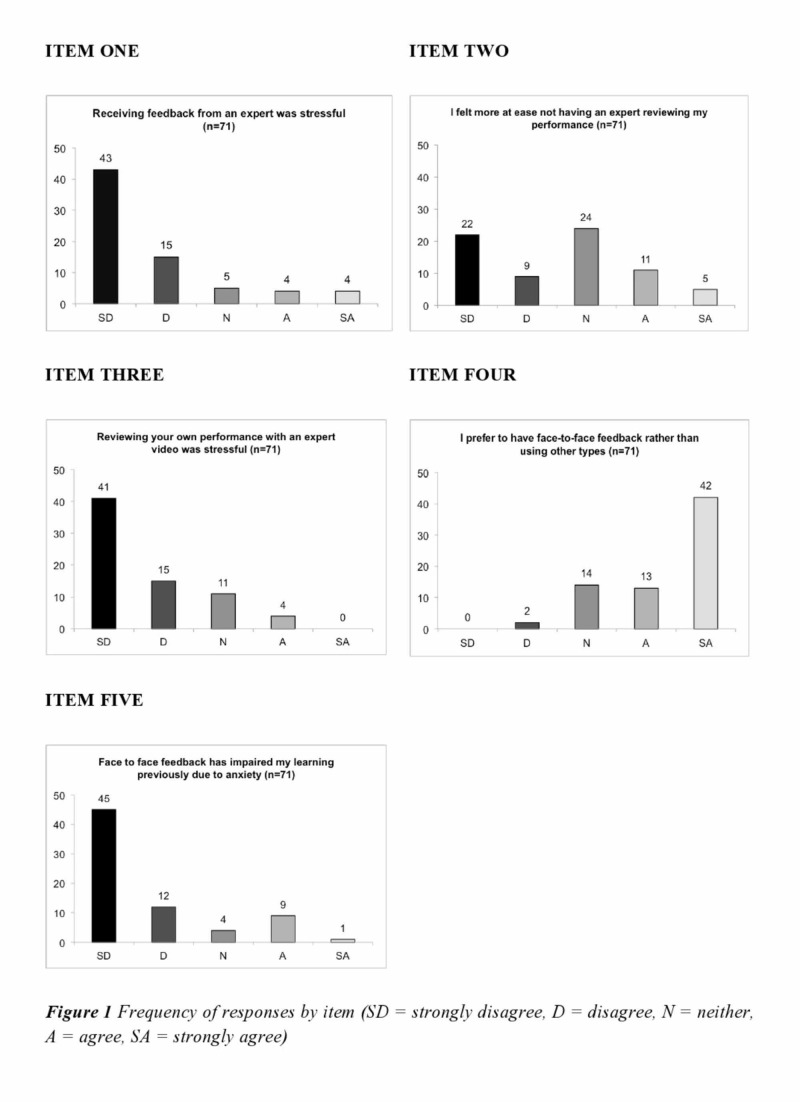
Responses to questionnaire by item

The frequency of responses and overall median response by item are detailed in Figure 1 and Table 1, respectively. The majority of respondents did not consider SIVF stressful, with 81.6% either strongly disagreeing or disagreeing with the statement ‘Receiving feedback from an expert was stressful’ (see Item One).

Reviewing performance

A third of respondents (33.8%) neither agreed nor disagreed that reviewing their performance without an expert (UGVF, see Item Two) was stressful, 44% disagreed with the statement. Around 78.9% of respondents disagreed or strongly disagreed with the statement that reviewing their performance with an expert video was stressful (see Item Three). Indeed, there was no significant difference found between the mean responses of Item One and Three (p=0.72).

Preferred feedback mechanism

Responses to Item Four indicated that the feedback method most preferred by students was SIVF, with over three-quarters (77.5%) of respondents in agreement with this statement.

Anxiety with face-to-face feedback

Around 79.1% of respondents either disagreed or strongly disagreed with the statement that face-to-face feedback had impaired their learning due to anxiety previously (see Item Five).

## Discussion

In 1984, Kolb proposed a model of learning that has since underpinned professional development and the concept of the ‘reflective practitioner’ prevalent in medical and surgical education [13]. According to this model, learning is experiential (‘learning by doing’) and occurs in a circular fashion; ideas form and change through individual experiences and are formed and modified through experiences. The shift in competency on the continuum from novice to expert results is aided by feedback received at various points in the learning cycle. This feedback should place emphasis on reflective practices to facilitate the progression of a given learner’s professional autonomy, being able to critically evaluate their own and others performances and by learning to self-monitor [14].

Although there remains much debate in the literature on the best method of feedback delivery, video feedback (VF) for developing technical clinical skills is a promising area. Current literature on the subject is somewhat confusing, with one study noting no significant difference when using VF for vascular anastomoses [15], and another two randomised trials that have, conversely, demonstrated significant improvements in practical skills acquisition when employing VF [16-17]. Students have also been shown to favour VF over generic and didactic lecture-based feedback in a questionnaire-based study [18].

A review of the literature suggests that acute stress can impede performance across a heterogenous group of clinical and simulated tasks [19]. In specific relation to VF, it has been previously suggested that anxiety may prevent full student benefit from VF sessions [20]. Whilst one multi-centre randomised study has shown reduced anxiety in relation to oral presentation skills when VF was used, there are no other studies demonstrating similar findings with VF [21]. Indeed, increased perceived anxiety associated with VF in the setting of non-technical skill acquisition has been reported [20, 22-23]. However, until now, no previous studies have investigated perceived anxiety levels in the context of practical skill acquisition when using VF.

Our study suggests that students both want and value feedback on procedural performance and prefer SIVF over UGVF. It also suggests that students experience minimal anxiety with either feedback modality and this is in contrast to Lindon-Morris and Laidlaw’s findings in a non-technical (communication) skills setting [20]. Several reasons may account for this. Firstly, Lindon-Morris and Laidlaw considered VF with tutor and peer group appraisal post-recording, whereas one-on-one private tutor-based feedback (SIVF) was considered in the current study. There is still no conclusive evidence as to the efficacy of giving feedback in a group setting or individually [24]. We hypothesise that the anxiety experienced by a student being ‘judged’ by their peers may be far greater than that experienced when obtaining feedback from a tutor in private. Secondly, our study considered procedural and surgical skills rather than non-technical skills. The steps required in undertaking a technical procedure are inherently more discrete, predictable and prescriptive than those for non-technical skills, such as history taking. Technical skills generally follow a standardised step-wise method and the predictability of the skills being reviewed in this study may confer a level of reassurance to students. Thirdly, there are several additional factors which were mitigated in our study but may have played an anxiety-provoking role in the Lindon-Morris and Laidlaw study: these include (1) public viewing of video performance, (2) lack of anonymity (recorded video in the current study only filmed the hands of participants), (3) personality traits evident (voice, facial expressions, mannerisms) and (4) lack of a clear step-wise set of responses to the scenario being performed.

The lack of consensus observed with regards to not having an expert review candidates' performance (Item 2) suggests that they did not find SIVF any more (or, indeed, any less) stressful than UGVF, which is in line with the results from Item 3. It is worth noting, however, that 16 participants (22.5%) strongly agreed that they were more at ease not having their performances reviewed by an expert, highlighting the incongruous nature of student preference in relation to feedback. This highlights the importance of diversity in feedback and that a ‘one-size-fits-all’ model of delivering feedback is unlikely to work. Learners tend to value feedback when someone they consider credible is providing it [24]; credibility is linked to either a previous relationship based on respect or on subject knowledge. In our study, participants did not have any previous relationship with any of the experts and would have been unlikely to be able to gauge their expert’s level of experience, thus lessening the perceived credibility of the experts to provide feedback.

Although SIVF proved popular amongst this cohort of students, its practical application into clinical practice is limited by resource-allocation to medical and surgical education; time constraints, tutor availability and the financial costs of individualizing the feedback process cannot be overstated. Conversely, UGVF has been purported to be a cost-effective and pragmatic solution for larger cohorts, with no significant differences found in terms of educational efficacy previously [12, 17-18]. Although the initial setup of UVGF educational material is undoubtedly a time and resource-consuming endeavour, once produced, these materials could theoretically be used for an unlimited number of students. Not only could this form of feedback minimise subsequent financial and temporal costs associated with delivery of effective feedback, it could also help students develop a more reflective, self-assessment based approach to learning, a key element of the feedback process.

Overall, given the findings of the current study, anxiety can tentatively be discounted as a confounder when the educational efficacy of SIVF and UGVF are compared, particularly when an effort to minimise other anxiety-provoking variables (i.e., those linked with non-technical skills) have been eliminated. However, it must be acknowledged that a large number of students preferred tailored SIVF, illustrating some of the complexities of balancing efficacy, resources, costs and learner preference when providing feedback. 

There are some limitations to this study. The participants of this study were volunteers who were unaware that the study would involve video recording and review of their performance. This perhaps means these students were a self-selecting and highly motivated group who would be keen on receiving feedback as they were eager to develop and learn these new skills. They were initially unaware that the study would involve video feedback to mitigate against the risk of a self-selection bias, where only students who are comfortable being video-recorded volunteered. The option of not participating was given to students after the trial was outlined to them; however, no participants opted to withdraw, suggesting that video recording was not a major concern for students. In a recent review of the literature, a tentative recommendation was made to utilise video review in conjunction with feedback as a component of learning and teaching [24]. 

A further limitation is the manner and approach of the tutor in the supervised group. However, a pre-study briefing was performed to try and ensure a degree of consistency in how feedback was provided. 

Participants knew this trial did not contribute to their assessed course, was not linked to any formative or summative assessment and the feedback was provided purely for their own learning. Feedback linked formally with the undergraduate medical course would be likely to evoke more anxiety than merely participating in a trial. Additionally, formal assessments of skills at this medical school are undertaken with an assessor present and this may be an additional anxiety-provoking element that our study has not assessed, and thus constituting a limitation.

The use of Likert questions is a restrictive modality of questioning and a follow-up study with a small sample of students, who could be interviewed in focus groups; using semi-structured interviews would provide a rich understanding of the numerous issues relating to anxiety levels using VF. In order to get a better grasp of which aspects of feedback they were particularly anxious about, and also how these anxieties could be potentially eased, interviews exploring these aspects would be potentially very beneficial. Additionally, the use of pre- and post-procedure anxiety scores could have enhanced our understanding of VF-related anxiety even further. 

## Conclusions

Whilst the limitations of this study are acknowledged, the findings are clear: in the context of simulated technical skills training, perceived anxiety is negligible and comparable in both video feedback modalities studied here. The use of a UGVF framework for simulated technical skills could enable graduating students to develop proficiency in core procedures in keeping with expectations of the GMC guidelines, whilst also mitigating institutional financial costs and educator time constraints.

Further study is required around the impact of different methods of simulation and feedback on student learning. In addition, a better understanding of the aspects of UGVF that made it a less popular modality for receiving feedback should be explored. This may help in addressing concerns, potentially making it more effective which would be of great benefit given the financial and time constraints that exist teaching students. 
